# Special Issue “Design, Synthesis, and Mechanism of Fluorescent and Luminescent Materials”

**DOI:** 10.3390/ijms26178198

**Published:** 2025-08-23

**Authors:** Leire Gartzia-Rivero, Jorge Bañuelos

**Affiliations:** Departamento de Química Física, Facultad de Ciencia y Tecnología, Universidad del País Vasco-EHU, Apartado 644, 48080 Bilbao, Spain; jorge.banuelos@ehu.eus

## 1. Introduction

Fluorescent and luminescent materials continue to revolutionize a broad spectrum of disciplines, including chemical sensing, biological imaging, lighting, displays, photodynamic therapy (PDT), and information storage. The field has evolved rapidly in recent years, driven by the convergence of synthetic chemistry, supramolecular design, materials science, and theoretical modeling. Key advances include the development of stimuli-responsive fluorophores, aggregation-induced emission (AIE) systems, room-temperature phosphorescent (RTP) materials, and upconversion nanomaterials, which enable precise control over brightness, lifetime, wavelength, and environmental sensitivity [1,2,3,4,5].

Since the seminal review by Tang et al. established AIE as a powerful paradigm to circumvent aggregation-caused quenching and harness solid-state luminescence [1], the field has diversified into multiple functional domains. Innovative strategies have been employed to fine-tune emission characteristics via molecular engineering (e.g., substitution patterns, donor–acceptor interactions, and rigidification), host–guest architectures (e.g., MOFs and zeolites), and multiscale modeling approaches that predict photophysical properties with increasing accuracy [6,7,8]. In parallel, new hybrid materials that combine luminescence with magnetic, redox, or biological activity have emerged, offering multifunctionality in sensors, diagnostics, and smart materials [9,10].

This Special Issue of the *International Journal of Molecular Sciences*, titled “Design, Synthesis, and Mechanism of Fluorescent and Luminescent Materials”, showcases recent contributions that exemplify this interdisciplinary progress. The selected articles span inorganic, organic, and hybrid materials, characterized with photonic and computational tools, illustrating how the precise design and structural control of luminophores can lead to new functional properties and mechanistic understanding.

## 2. Overview of Published Articles

### 2.1. Advances in Inorganic and Hybrid Luminescent Systems

Rudolph et al. introduce a new europium (II) hydride oxide iodide, Eu_5_H_2_O_2_I_44_, whose orthorhombic structure incorporates [HEu4]^7+7^ and [OEu4]^6+6^ tetrahedra. This compound exhibits blue-green photoluminescence centered at 463 nm, the shortest-wavelength emission reported so far for Eu^2++^ hydride-containing compounds. Additionally, it shows ferromagnetic behavior below ~10 K, making it a rare example of a bifunctional luminescent–magnetic material with potential applications in quantum sensing and data storage.

Oliden-Sánchez et al. explore how confinement effects within nanoporous aluminophosphate frameworks modulate the luminescent behavior of the styryl dye 4-DASPI. Two host structures with distinct topologies—channels and cavities—were employed. The authors demonstrate how host geometry and dye–framework interactions control emission color, quantum efficiency, and excited-state lifetimes, offering design principles for next-generation solid-state luminophores and optical sensors.

### 2.2. Functional Organic Systems and Photosensitizers

Díaz-Norambuena et al. examine a series of orthogonal BODIPY dimers modified via regioselective formylation. Through photophysical, electrochemical, and theoretical analysis, they demonstrate that formyl substitution tunes the interplay between fluorescence emission and singlet oxygen generation. This structure–activity relationship enables the rational tuning of excited-state pathways, making these systems promising for dual-function applications in phototheragnostics.

Kopcsik et al. report on 1-formamido-5-isocyanonaphthalene (ICNF), a luminescent derivative formed through the hydrolysis of 1,5-diisocyanonaphthalene. The molecule exhibits solvatochromic fluorescence, with significant emission shifts depending on solvent polarity and hydrogen bonding. Remarkably, it retains strong emission in aqueous environments, making it suitable for environmental and biological sensing. The work also contributes to understanding the stability and reactivity of isocyanide-based luminophores under ambient conditions.

### 2.3. Computational Design and Theoretical Insights

Piekos and Zadykowicz employ DFT and TD-DFT calculations to design acridinium-based chemiluminogens, key components in clinical diagnostics. The authors explore the structural modifications that influence light emission efficiency, lifetime, and chemical reactivity. Their work identifies new derivatives with enhanced chemiluminescent potential, guiding future experimental synthesis. This study illustrates how computational screening is becoming a powerful tool for accelerating discovery and optimization in luminescent materials research.

## 3. Conclusions

The contributions to this Special Issue collectively underscore the strategic interplay between molecular design, supramolecular organization, and theoretical insight in advancing luminescent materials. A recurring theme across the studies is the fine-tuning of excited-state dynamics through subtle variations in molecular architecture, such as substitution patterns and rigidification strategies, which enable control over emission wavelengths, lifetimes, and quantum yields. Equally significant is the role of host–guest interactions and nanoscale confinements in regulating luminescent behavior and stabilizing otherwise labile excited states. In this regard, computational methods are gaining increased recognition as a tool for predicting photophysical properties, accelerating rational design, and reducing trial and error in synthesis. Collectively, these studies provide a roadmap for developing multifunctional luminescent materials with tailored properties for applications ranging from sensing and imaging to data storage and phototherapy.
